# Supplementary material for: Effect of short inter-pregnancy interval on perinatal and maternal outcomes among pregnant women in SSA 2023: Systematic review and meta-analysis
Source: PLoS One. 2025 Jan 7;20(1):e0294747. doi: 10.1371/journal.pone.0294747 (PMC11706456; doi:10.1371/journal.pone.0294747)
Supplement: Attachment — Submitted filename: review comment.docx [file pone.0294747.s001.docx]

Paper review
**abstract**
overall very descriptive
**introduction**
please add more studies about your topic in introduction section

**method**

very good you need to justify in detailed your quality assessment and what criteria you used.
**results**
very good

**Discussion**
very good
